# Functional and anatomical outcomes of brolucizumab for nAMD in a real-life setting

**DOI:** 10.1038/s41598-024-51315-0

**Published:** 2024-01-16

**Authors:** Marco Rocco Pastore, Serena Milan, Gabriella Cirigliano, Daniele Tognetto

**Affiliations:** Eye Clinic, Department of Medicine, Surgery and Health Sciences, University of Trieste, Trieste, Italy

**Keywords:** Drug discovery, Medical research

## Abstract

To report long-term outcomes of brolucizumab in neovascular age-related macular degeneration (nAMD) treatment. Records from 74 patients were retrospectively reviewed. Both naïve eyes and those previously treated with other antiVEGF agents were included. Primary outcomes included variation in best corrected visual acuity (BCVA), central subfield thickness (CST), intraretinal fluid (IRF), subretinal fluid (SRF), and pigment epithelial detachment (PED) dimensions. Outcomes were reviewed after the loading phase, at week 24, and at last follow-up. IOI occurrence represented the secondary outcome. BCVA improved significantly in both groups. In switched eyes, IRF and SRF were significantly reduced at every timepoint, with CST reduction from week 24 (p = 0.005). In naïve group, CST decreased from the loading phase (p = 0.006) and all patients showed dry macula from week 24. A significant reduction in PED maximum high was demonstrated in both groups. In seven naïve eyes, PED completely reabsorbed; a slight increase in PED horizontal maximal diameter was also observed from week 24. IOI occurred in 5.4% of cases. In conclusion, brolucizumab showed a strong drying effect, permitting functional improvement together with fluid reabsorption and an encouraging modification of PED dimension, especially on naïve patients. These results together with the extension of treatment intervals make brolucizumab an efficient therapeutic strategy for nAMD.

## Introduction

Brolucizumab represents one of the latest innovations for the treatment of neovascular age-related macular degeneration (nAMD). It is a 26 kDa humanized single-chain variable fragment antibody that inhibits all vascular-endothelium-growth-factor-A (VEGF-A) isoforms^1^. It was first approved for the treatment of nAMD in October 2019 and in February 2020 respectively by the FDA and the EMA based on phase 3 HAWK and HARRIER clinical trials results^2,3^. In the results best-corrected visual acuity (BCVA) with brolucizumab treatment (dosed every 12 or 8 weeks after a loading phase of 3 monthly intravitreal injections—IVI) was non-inferior to aflibercept (administered every 8 weeks after a loading phase of 3 monthly IVI). Functional improvement was accompanied by considerable macular structural modifications: brolucizumab treatment was associated with a greater central subfield thickness (CST) reduction and a more efficient fluid control. Another advantage is represented by the needing for less frequent IVI, potentially scheduled with a 12-weeks interval, reducing treatment burden, for both patients and physicians. Despite the great enthusiasm, concerns about its clinical use arose because of the increasing number of post-marketing reports of intraocular inflammation (IOI) and vascular occlusion^4^. A post-hoc analysis of the study HAWK and HARRIER was required, and the investigators reported an IOI incidence of at least 4.6% in eyes treated with 6 mg brolucizumab. This event typically occurs within the first 3 months of therapy and can be associated with retinal vasculitis, and retinal artery occlusion, with poor prognosis^4^. The present study aims to evaluate anatomical and functional outcomes with brolucizumab for nAMD treatment in a European tertiary referral center.

## Methods

This is a retrospective observational study at the Eye Clinic, University of Trieste, Italy. Clinical records of nAMD patients treated with brolucizumab IVI (IVB), 6 mg, in routine clinical practice between March 2021, and September 2023, at our Institution, were reviewed. Inclusion criteria were a diagnosis of nAMD with any type of macular neovascularization (MNV) as the primary indication for treatment; a minimum of 3 IVB; at least 6 months of follow-up after the first IVB. Only patients with monolateral lesion were included. Both anti-VEGF-naïve eyes and eyes already treated were enrolled^4^. For treatment-naïve patients, brolucizumab was proposed following an extensive discussion of the risks and benefits involved. For patients already receiving IVI, the decision to switch to brolucizumab was proposed based on the response to previous IVI according to Amoaku et al. criteria^5^. Exclusion criteria were: concomitant ophthalmological diseases (e.g., diabetic retinopathy, glaucoma, chorioretinal diseases); laser macular photocoagulation or photodynamic therapy; media opacities; previous history of IOI; autoimmune disease. Before starting the treatment, all patients underwent complete ophthalmological examinations, including BCVA assessment, intraocular pressure measurement, slit-lamp biomicroscopy, Spectral-Domain-Optical-Coherence-Tomography (SD-OCT), and OCT-angiography (OCT-A), fluorescein-angiography (FA) and indocyanine-green-angiography (ICGA). SD-OCT, OCT-A, FA and ICGA were obtained with Heidelberg Spectralis II (Software Version 6.15, Heidelberg Engineering, Heidelberg, Germany). NAMD diagnostic criteria were based on a previous report of nAMD nomenclature^6^.The day of the first IVB was considered as the baseline follow-up. In case of IOI, treatment was promptly interrupted, and FA was performed. Furthermore, patients were educated about IOI symptoms and advised to report immediately if any adverse events were noted^7^. Primary outcomes including variation in CST, intraretinal fluid (IRF), subretinal fluid (SRF), and pigment-epithelial-detachment (PED) were evaluated at baseline (T0), after the loading phase (T1), at week 24 (T2), and at last follow-up (T3). BCVA was evaluated at T0 and T3. The number of injections was also recorded^8^. CST was defined as the average thickness between the internal limiting membrane (ILM) and Bruch’s basal membrane (BM) within the central 1 mm of the fovea^9^. PED was defined as the separation between the retinal pigment epithelium and BM. PED-horizontal-maximal-diameter (PED-HMD) and PED-maximum-high (PED-MH) were manually measured using the caliper tool available on on Heidelberg Spectralis II (Software Version 6.15, Heidelberg Engineering, Heidelberg, Germany)^10^. All measurements were assessed by two independent retinal specialists and compared using the Cohen’s kappa coefficientDry macula was defined when no macular IRF, SRF, sub-RPE-fluid, hemorrhages, or exudation was detected^7^. BCVA was evaluated using an Early Treatment of Diabetic Retinopathy Study (ETDRS) chart at 4 m distance and reported as ETDRS letters equivalent (where a BCVA of 20/20 was defined as 85 ETDRS letters) for the statistical analysis. The secondary outcome was IOI occurrence, focusing on symptoms, signs, time to the event from the first IVB and the most recent IVB, number of IVB to the event, and visual outcome at resolution. In switched patients, the first 3 injections were administered every 6 weeks; the following 2 every 10 weeks; then intervals were scheduled based on OCT evidence: in case of resolved IRF, and resolved or stable SRF, they were extended by 2 weeks; if not, they were reduced to 8 weeks. In treatment-naive patients, after the loading phase (3 monthly IVB), injections were administered at every-8-weeks/every-12-weeks intervals according to disease activity based on previously mentioned OCT evidence as for switched patients.

Descriptive analysis was performed using mean and standard deviation (SD) for the continuous variables and percentage values for the categorical variables. Paired T-test was used to assess mean CST differences between T0 and T1, T0 and T2, and T0 and T3, and to analyze mean BCVA differences between T0 and T3. McNemar test for dependent proportions was applied to assess differences in the percentage of unresolved patients, meaning patients with residual IRF or SRF. All statistical analyses were performed using R Statistical Software (version 3.5.3; R Foundation for Statistical Computing, Vienna, Austria). All tests were two-tailed, and a statistical significance was defined as a p-value < 0.05. The study protocol adhered to the tenets of the Declaration of Helsinki and was approved by the Ethics Committee of the University of Trieste (107/22, 19 October 2022). The nature and the purpose of the investigation were fully explained, and written informed consent was obtained from all participants.

## Results

### Demographic analysis

74 eyes of 74 patients met the inclusion criteria and were included in the analysis. Demographic analysis is represented in Table 1. Four eyes (5.4%) of 4 patients developed IOI and were thus excluded from further analysis. Table 2 illustrates IOI features. As regards the switched group, 29 out of 54 patients were males (53.7%); the mean number of other anti-VEGF agents IVI per eye was 19.98 ± 9.74; the average follow-up time was 49.7 weeks (range 38–105); the mean number of IVB performed was 6.1 (range 5–11) per eye; the average injection interval was 7.9 weeks (range 6–12). As regards the naïve group, 10 out of 16 patients were males (62.5%); the average follow-up time was of 48 weeks (range 36–60 weeks); over the study time, the mean number of IVB performed was 6.4 (range 5–8) per eye. The average injection interval was 7.6 weeks (range 4–12). The interobserver agreement on image analysis was k = 0.92 (p < 0.01).

**Table 1:** Demographic characteristics of the study cohort.

Characteristics	
Patients, n (eyes, n)	74 (74)
Sex (male:female)	41:33
Treatment status (naïve:switched)	17:57
Mean age ± SD (years)	79.34 ± 2.83
Comorbilities, n of patients	
None	18
Hypertension	27
Cardiovascular disease	13
Diabetes	10
Allergy	12
MN Vtype, n of eyes (naïve:switched)	
Type 1	37 (8:29)
Type 2	23 (6:17)
RAP	3 (0:3)
PCV	5 (3:2)
Mixed	6 (1:5)
Weeks of follow up (months), n of patients	
36 (9)	3
38 (9.5)	6
42 (10.5)	2
48 (12)	35
54 (13.5)	5
60 (15)	10
72 (18)	3
84 (21)	3
≥ 92 (≥ 23)	3

*SD* standard deviation, *MNV* macular neovascularization, *RAP* retinal angiomatous proliferation, *PCV* polypoidal choroidal vasculopathy.

**Table 2:** Brolucizumab related intraocular inflammation occurrence.

Outcome	Patient 1	Patient 2	Patient 3	Patient 4
Previous antiVEGF injections, n	23	16	Naive	16
Previous antiVEGF agents, n	2	3	–	3
Sex	Female	Male	Female	Male
Comorbility	None	Diabetes	Hypertension	Diabetes
IVB to event, n	2	4	3	2
Time to event from the first IVB, days	48	164	130	84
Time to event from most recent IVB, days	19	15	15	42
Symptoms	Floaters	Floaters	Floaters	VA impairment
BCVA at baseline	20/125	20/50	20/200	20/160
BCVA after inflammation resolution	20/100	20/50	20/100	20/200
Inflammation	Vitritis	Vitritis	Vitritis	Vitritis
				Vascular occlusion*

*AntiVEGF* anti-vascular endothelial growth factor, *IVB* intravitreal injection of brolucizumab, *BCVA* best corrected visual acuity.

*Vascular occlusion was detected only in patient number 4.

### Functional and anatomic outcomes

#### Switched group

**Figure 1:**
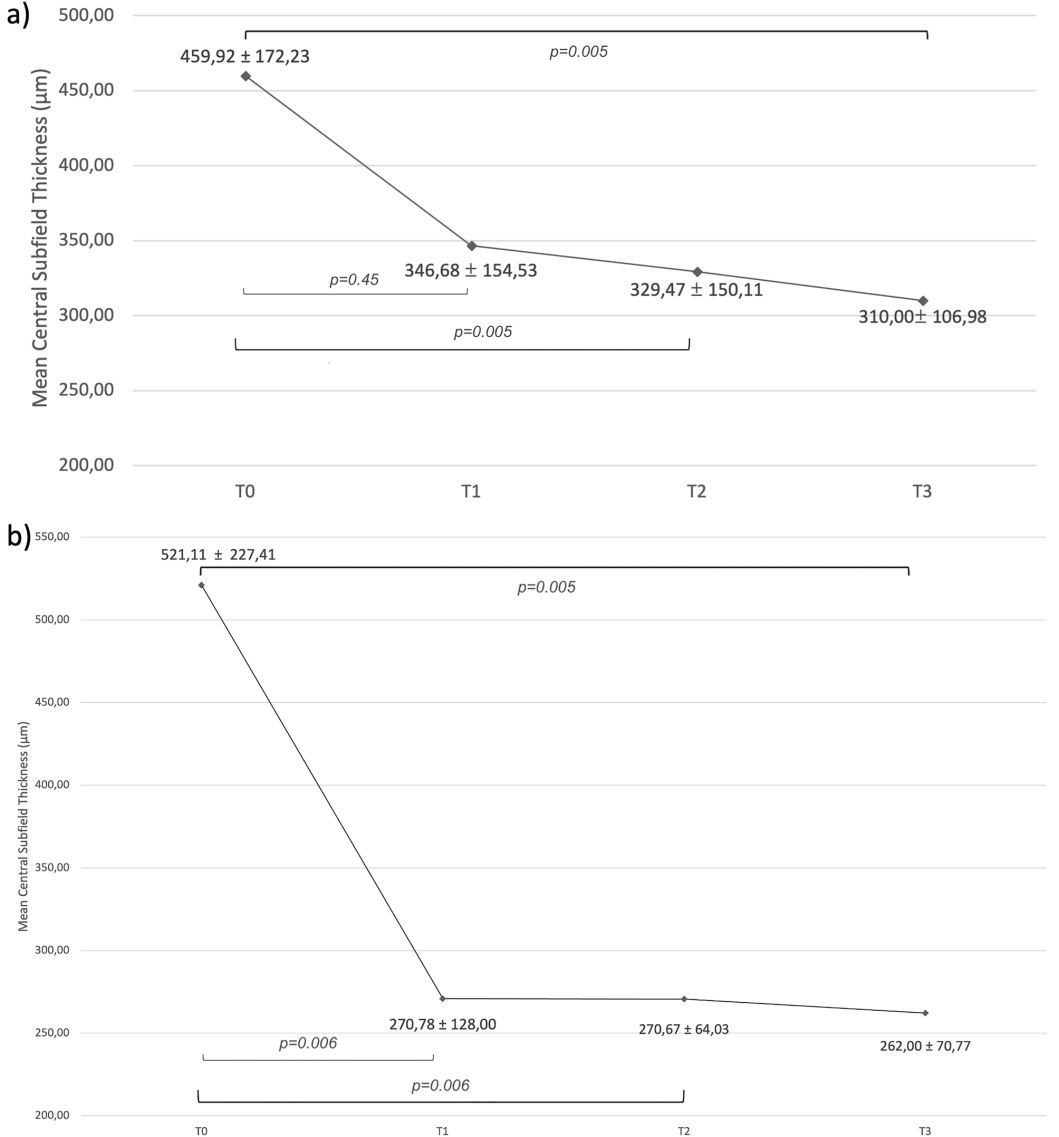
Central Subfield Thickness variation’s trend in switched patients (**a**) and naïve patients (**b**) at different time points. 70 patients were analyzed, 54 in the switched group, 16 in the naïve group. Thickness is shown in the y-axis (values are expressed in μm), timepoints in the x-axis (T0 = baseline; T1 = after the loading phase; T2 = at week 24; T3 = at last follow-up). Mean values ± standard deviation are reported at each timepoint. Statistical significance was defined as a p-value < 0.05. Significance *(p)* between T1–T0, T2–T0, T3–T0 is reported under brackets: statistical reduction was noted between T2–T0 and T3–T0 in switched patients and at all timepoints in naive patients.

**Figure 2:**
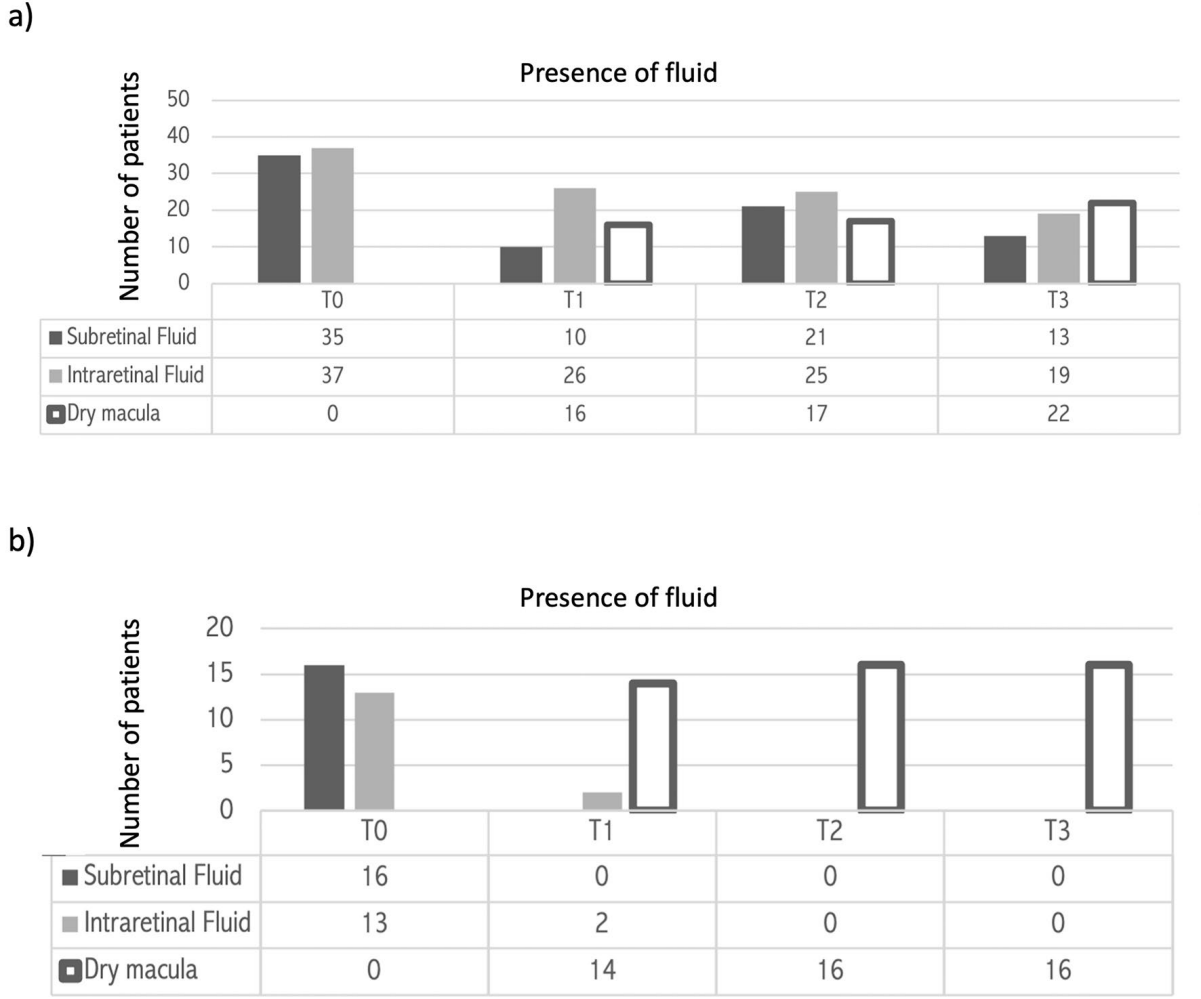
Presence of subretinal fluid and intraretinal fluid in switched patients (**a**) and naïve patients (**b**) at different time points. 70 patients were analyzed, 54 in the switched group, 16 in the naïve group. Number of patients affected by any kind of fluid is shown in the y-axis, timepoints in the x-axis (T0 = baseline; T1 = after the loading phase; T2 = at week 24; T3 = at last follow-up). Dark grey columns represent subretinal fluid; light grey columns represent intraretinal fluid; white columns represent dry macula. In both study groups, the number of patients presenting any kind of fluid was statistically reduced at every timepoint compared to T0 (p < 0.05), with consequent increase in the number of patients with dry macula.

**Figure 3:**
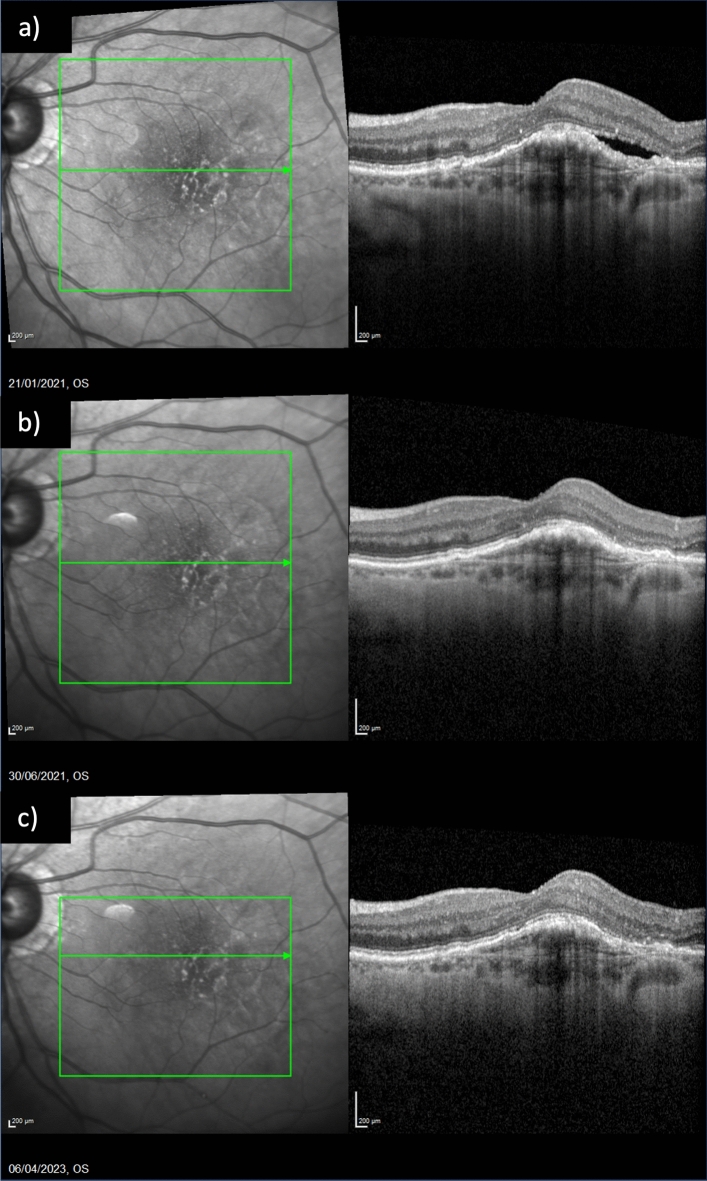
Fluid control in a patient already treated with eighteen injections of three different antiVEGF agents. (**a**) Exudation at baseline; (**b**) subretinal fluid resolution after one injection of Brolucizumab; (**c**) maintenance of the result at last follow up, after eleven injections of Brolucizumab.

**Figure 4:**
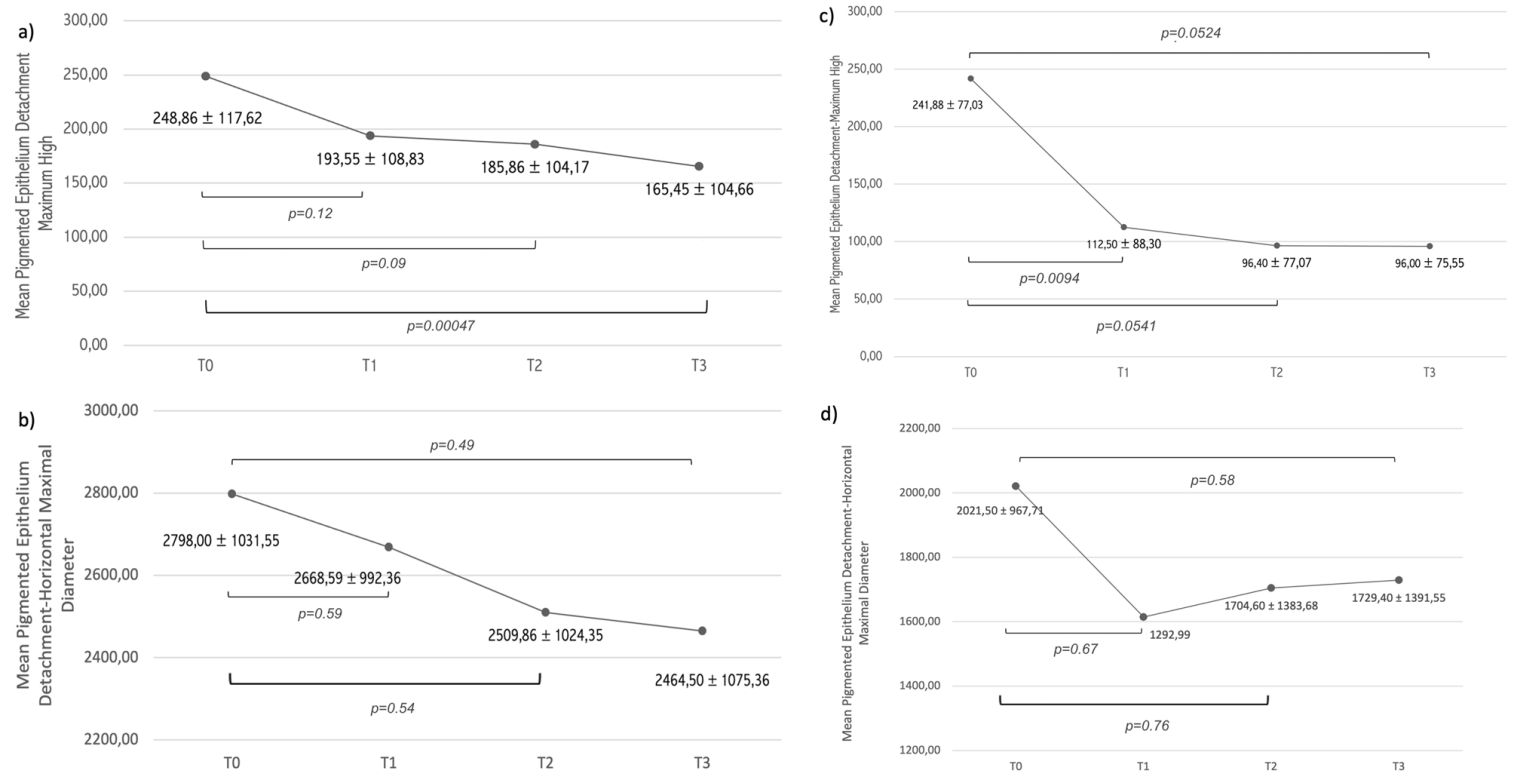
Pigmented epithelium detachment dimensions’ variation at different time points. (**a**) Mean maximal high variation in switched patients; (**b**) mean horizontal maximal diameter variation in switched patients. (**c**) Mean maximal high variation in naive patients; (**d**) mean horizontal maximal diameter variation in naive patients. 70 patients were analyzed, 54 in the switched group, 16 in the naïve group. Dimension is shown in the y-axis (values are expressed in μm), timepoints in the x-axis (T0 = baseline; T1 = after the loading phase; T2 = at week 24; T3 = at last follow-up). Statistical significance was defined as a p-value < 0.05. Significance *(p)* between T1–T0, T2–T0, T3–T0 is reported under brackets. Mean Pigmented Epithelium Detachment Maximal High statistically reduced in switched patients between T3–T0, in naïve patients at all timepoint. No statistically significant modification in Mean Pigmented Epithelium Detachment Horizontal Maximal Diameter was noted neither in switched patients nor in naïve patients.

**Figure 5:**
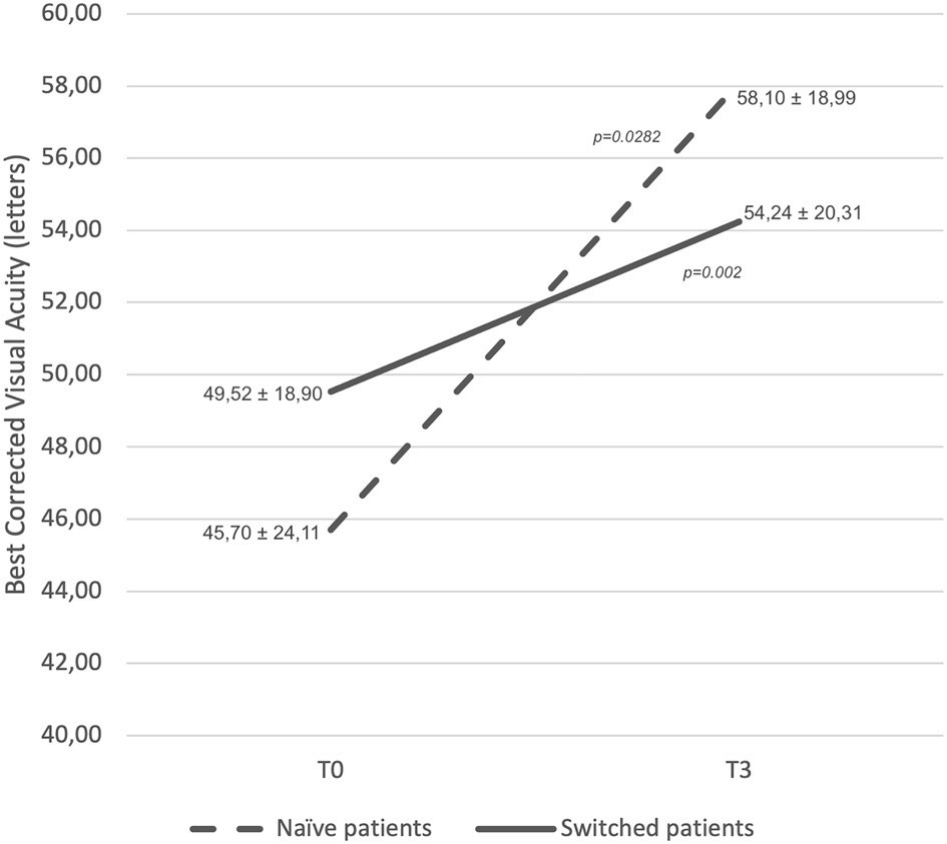
Best corrected visual acuity variation in switched patients (solid line) and naïve patients (dashed line) between T0 and T3. 70 patients were analyzed, 54 in the switched group, 16 in the naïve group. Best corrected visual acuity is shown in the y-axis (values are expressed in letters), timepoints in the x-axis (T0 = baseline; T3 = at last follow-up). Mean values ± standard deviation are reported at each timepoint. Statistical significance was defined as a p-value < 0.05. Significance *(p)* between T0 and T3 is reported under brackets: statistical improvement was noted in both switched and naïve patients.

Mean CST was 459.92 ± 172.23 μm (range 294–1011 μm) at T0, 346.68 ± 154.53 μm (range 209–700 μm) at T1, 329.47 ± 150.11 μm (range 150–700 μm) at T2 and 310.00 ± 106.98 μm (range 224–563 μm) at T3. The CST variation trend is represented in Fig. 1a*.* CST reduced between T0 and T2 (p = 0.005) and the significant result was confirmed at T3. The presence of SRF and IRF at different time points is summarized in Fig. 2a. SRF was present in 35 (68.6%) patients at T0, 10 (19.6%) patients at T1, 21 (41.2%) patients at T2, and 13 (25.5%) patients at T3. SRF was reduced between T0 and T1 (p < 0.00001), T0 andT2 (p = 0.00528), and T0 and T3 (p < 0.00001). IRF was present in 37 (72.5%) patients at T0, 26 (51.0%) patients at T1, 25 (49.0%) patients at T2, and 19 (37.3%) patients at T3. IRF reduced between T0 and T1 (p = 0.0251), T0 and T2 (p = 0.00034) and T0 and T3 (p = 0.0151). Dry macula was noted in 16 (31.4%) patients at T1, 17 (33.3%) patients at T2, and 22 (43.1%) patients at T3 (Fig. 3). Mean PED-MH was 248.86 ± 117.62 μm at T0, 193.55 ± 108.83 μm at T1, 185.86 ± 104.17 μm at T2 and 165.45 ± 104.66 μm at T3 (shown in Fig. 4a). A statistical difference was found between T0 and T3 values (p = 0.00047). Mean PED-HMD (shown in Fig. 4b) was 2798.00 ± 1031.55 μm at T0, 2668.59 ± 992.36 μm at T1, 2509.86 ± 1024.35 μm at T2 and 2464.50 ± 1075.36 μm at T3. No statistical difference was demonstrated. Complete PED resolution wasn’t observed in any patient. Baseline mean BCVA was 49.52 ± 18.90 letters (20/100); it increased to 54.24 ± 20.31 letters (20/80) at T3 (p = 0.002), with a mean gain of 4.71 ± 6.06 letters (shown in Fig. 5). Results are summarized in Table 3.

**Table 3:** Anatomical and functional results.

Outcome	T0	T1	p (T1–T0)	T2	p (T2–T0)	T3	p (T3–T0)
Switched patients							
BCVA (letters), mean ± SD	49.52 ± 18.9	–	–	–	–	54.24 ± 20.31	**0.002**
CST, mean (range)	459.92 ± 172.23 (294–1011)	346.68 ± 154.53 (209–700)	*0.45*	329.47 ± 150.11 (150–700)	**0.005**	310.00 ± 106.98 (224–563)	**0.005**
SRF, n of pts (%)	35 (68.6%)	10 (19.6%)	** < 0.00001**	21 (41.2%)	**0.00528**	13 (25.5%)	** < 0.00001**
IRF	37 (72.5%)	26 (51.0%)	**0.0251**	25 (49.0%)	**0.00034**	19 (37.3%)	**0.0151**
Dry macula, n of pts (%)	0	16 (31.4%)	–	17 (33.3%)	–	22 (43.1%)	–
Mean PED-MH	248.86 ± 117.62	193.55 ± 108.83	*0.12*	185.86 ± 104.17	*0.09*	165.45 ± 104.66	**0.00047**
Mean PED-HMD	2798.00 ± 1031.55	2668.59 ± 992.36	*0.59*	2509.86 ± 1024.35	*0.54*	2464.50 ± 1075.36	*0.49*
Naïve patients							
BCVA (letters)	45.70 ± 24.11	–	–	–	–	58.10 ± 18.99	**0.0282**
CST, mean (range)	521.11 ± 227.41 (348–970)	270.78 ± 128.00 (210–631)	**0.006**	270.67 ± 64.03 (230–400)	**0.006**	262.00 ± 70.77 (196–380)	**0.005**
SRF, n of pts (%)	16 (100%)	0	** < 0.00001**	0	** < 0.00001**	0	** < 0.00001**
IRF	13 (81.3%)	2 (12.5%)	**0.0001**	0	** < 0.0001**	0	** < 0.0001**
Dry macula, n of pts (%)	0	14 (87.5%)	–	16	–	16	–
Mean PED-MH	241.88 ± 77.03	112.50 ± 88.30	**0.0094**	96.40 ± 77.07	**0.0541**	96.00 ± 75.55	**0.0524**
Mean PED-HMD	2021.50 ± 967.71	1614.88 ± 1292.99	*0.67*	1704.60 ± 1383.68	*0.76*	1729.40 ± 1391.55	*0.58*

*BCVA* best corrected visual acuity, *SD* standard deviation, *CST* central subfield thickness, *SRF* subretinal fluid, *IRF* intraretinal fluid, *PED-MH* pigment epithelium detachment-maximum-high, *PED-HMD* pigment epithelium detachment- horizontal-maximal-diameter.

Significant values are in bold and italics.

#### Naïve group

**Figure 6:**
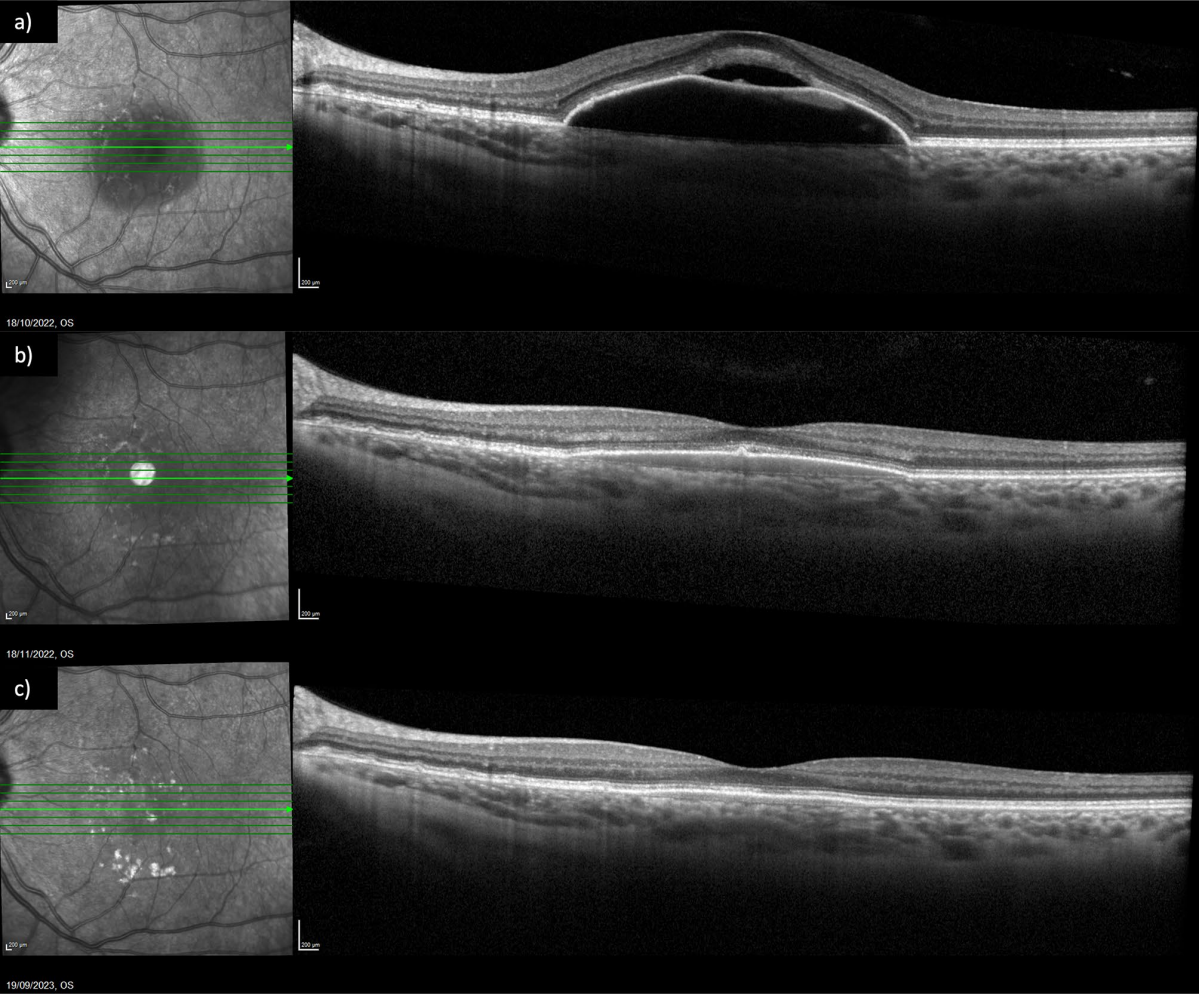
Fluid control in a naïve patient. (**a**) Exudation at baseline; (**b**) dry macula after one injection of Brolucizumab; (**c**) maintenance of the result at last follow up, after six injections of Brolucizumab on Q12.

Mean CST was 521.11 ± 227.41 μm (range 348–970 μm) at T0, 270.78 ± 128.00 μm (range 210–631 μm) at T1, 270.67 ± 64.03 μm (range 230–400 μm) at T2 and 262.00 ± 70.77 μm (range 196–380 μm) at T3. The CST variation trend is represented in Fig. 1b*.* CST reduced between T0 and T1 (p = 0.006) and the significant result was confirmed at all time points. The presence of IRF and SRF at different time points is summarized in Fig. 2b. SRF was present in 16 (100%) patients at T0; it completely resolved in all patients at T1 (p < 0.00001) and this result was maintained at T2 and T3. IRF was present in 13 (81.3%) patients at T0 and in 2 (12.5%) patients at T1 (p = 0.0001). A complete resolution of IRF was observed at T2 and this result was maintained at T3. Dry macula was observed in 14 (87.5%) patients at T1 and in all patients at T2 and T3 (Fig. 6). Mean PED-MH (shown in Fig. 4c) was 241.88 ± 77.03 μm at T0, 112.50 ± 88.30 μm at T1, 96.40 ± 77.07 μm at T2 and 96.00 ± 75.55 μm at T3. A statistical reduction was noted comparing T0 with T1 (p = 0.0094), T2 (p = 0.0541) and T3 (p = 0.0524). Mean PED-HMD (shown in Fig. 4d) was 2021.50 ± 967.71 μm at T0, 1614.88 ± 1292.99 μm at T1, 1704.60 ± 1383.68 μm at T2 and 1729.40 ± 1391.55 μm at T3. No statistical difference was noted between any timepoint. At T1, PED completely resolved in 4 eyes and the result was maintained at T2; it resolved in 3 additional eyes at T2; results were maintained at T3. Baseline mean BCVA was 45.70 ± 24.11 letters (20/125); it increased to 58.10 ± 18.99 (20/80) at last follow-up (p = 0.0282), with a mean gain of 12.4 ± 15.03 letters (shown in Fig. 5). Results are summarized in Table 3.

## Discussion

The present study reported our real-life experience with intravitreal brolucizumab for the management of nAMD patients, analyzing anatomical and functional status.

As regards structural results, in the naïve cohort, a significant reduction in CST was noted after the first three IVB. Dry macula was observed in 87.5% of patients at T1 and all patients at T2. SRF completely resolved in all patients at T1, while complete reabsorption of IRF was observed at T2. PED completely resolved in 7 out of 16 eyes.

Also in our switched subgroup, significant CST reduction was achieved starting from T2. Complete fluid reabsorption was possible also in this cohort: 31.4% of patients showed dry macula after the loading phase, increasing to 43.1% at T3. SRF fluid was detected in 35 patients (68.6%) at baseline and in 13 (25.5%) patients at the last follow-up. IRF was present in 37 (72.5%) patients at baseline and 19 (37.3%) patients at the last follow up. Complete PED resolution wasn’t observed in any patient.

Fluid presence constitutes a hallmark of active disease in nAMD and a threat to functional outcomes^11^. Persistent IRF correlated with macular atrophy in both the CATT and HARBOR studies^12^. Evans et al.^13^ demonstrated that lower thickness oscillation was associated with better BCVA and a lower tendency to develop macular fibrosis and geographic atrophy. Chakravarthy et al.^14^ showed that greater visual improvement is achieved when no IRF/SRF is detected at more than 2 clinic visits. Despite the revolutionary role of previous anti-VEGF medications, fluid is frequently detected in patients under intensive treatment^12,15^, representing a challenge for upcoming therapies. Brolucizumab showed promising results in phase 3 HAWK and HARRIER clinical trials: it was associated with a greater CST reduction compared to aflibercept. Moreover, its drying effect tended to be more stable over time, according to Sharma et al.^16^. Real-life data seem to confirm this great potential^17–20^.

In the treatment-naïve cohort of the REBA study, the loading phase determined exudation resolution in 76.0% of eyes^17^. Our naïve eyes showed more encouraging results: a higher percentage of patients (87.5%) achieved dry macula at T1 and 100% at T2, demonstrating the brolucizumab efficacy in fluid reabsorption, starting at the loading phase and maintaining the result over time. In the REBA study, only patients affected by type 1, type 2, or mixed MNV were analyzed, while in our naïve cohort, we also analyzed polypoidal choroidal vasculopathy: this may explain the different results. Fukuda et al., recently demonstrated that polypoidal lesions are particularly sensitive to brolucizumab, which determined a higher rate of complete lesion resolution compared to aflibercept^20^. It would be useful to evaluate the relationship between the type of lesion and the response to treatment: studies including a homogeneous number of different lesions’ types are required. There is similar evidence of brolucizumab efficacy in reducing CST and IRF/SRF since the very first injection in switched patients^3,10,19,20^. Conversely, we noted a sort of delay in fluid response in this subgroup. It is interesting to note that the loading phase wasn’t sufficient in determining a statistical reduction of CST in our subgroup. It may be related to the fact that most of the eyes had a longstanding history of treatment as demonstrated by the high mean number of other anti-VEGF agents’ injections per eye (19.98 ± 9.74). However, dry macula was obtained in 31.4% of patients after the loading phase, increasing to 43.1% at T3. It is notable if we consider that this group of patients was refractory to previous anti-VEGF agents and may present cystic alterations sometimes considered degenerative features and not susceptible to further treatment.

In both groups, fluid localization influenced its response to treatment. SRF seemed to be particularly responsive to brolucizumab with respect to IRF. This is in accordance with previous literature. Mishra et al. demonstrated that both IRF and SRF were detected in 82.1% of patients at baseline; after the loading phase, they reduced respectively to 41.1% and 16.1%^22^. Toto et al.^18^ showed a reduction in IRF and subfoveal-SRF rates in type 1 MNV between baseline and week 12, respectively changing from 41.7 to 20.8% and from 62.5 to 4.2%. The greater amount of unresponsive fluid in the intraretinal compartment may represent a biomarker of degenerative cystoid processes^23^.

We also analyzed PED modification, focusing both on PED-MH and PED-HMD. To our knowledge, there is currently no study evaluating this feature over such a long follow-up.

As regards naïve patients, we showed a statistical reduction of PED-MH after the loading phase. This is in accordance with Toto et al.^18^, who demonstrated a significant reduction between baseline and week 16 in naïve type 1 MNV. Thanks to our longer follow-up, the maintenance of the statistical result at week 24 and the last follow-up could also be demonstrated. In addition, we also evaluated PED-HMD: it reduced after the loading phase, with a slight increase at T2 and the last follow-up. 7 patients obtained a complete resolution of PED.

As regards switched patients, both PED-MH and PED-HMD progressively reduced, but only the former was statistically significant at the last follow. Any case of complete resolution of PED occurred. Previous studies on switched patients showed similar results. Rispoli et al.^10^ evaluated PED-HMD and PED-MH variations 1 month after the first IVB: both reduced, with statistical results in PED-MH. The BREW study^19,21^ analyzed a series of 42 switched eyes with a mean follow-up of 7.2 ± 3.6 weeks: PED was present in 31 eyes at baseline and resolved in 2 eyes, reduced in 13, and remained stable in 16.

In our series, PED was shown to be less responsive to anti-VEGF treatment compared to other fluid locations, probably because of its double composition (i.e., fibrovascular and fluid)^24^. MNV can be defined as immature and mature according to vascular network morphology. The former has a high branching index and a high number of anastomoses, while the capillary fringe is lost in the latter, presenting larger vessels. Pruning of small capillaries and expansion of vessel caliber was related to IVI in nAMD^25^. Faes et al.^26^ demonstrated that mature MNVs were associated with a more frequent IVI regimen and a higher median number of total IVI. Mature MNVs are supposed to be supported by a rich extracellular milieu supplying local VEGF, making them more dependent on these local survival signals for sustenance. These aspects may explain why switched patients were less prone to regression with anti-VEGF treatment^25^. According to Serra et al.^27^, PED modification may be a useful marker of quiescent MNV conversion into active MNV: higher PED-MH is associated with a greater potential of conversion into active lesions, while a preferential increase in PED linear diameter is shown by not exudating lesions. In our series, a progressive reduction in PED-MH was noted in both groups. As regards PED-HMD, it reduced in switched patients; in naïve patients, an initial reduction was followed by a tendency towards an increase at the last follow-up. Dry macula was detected in all naïve patients at week 24 and fluid didn’t recur at the last follow-up: the increase in PED-HMD could probably indicate a conversion into a not exudative MNV.

As regards functional results, a mean gain of 12.4 ± 15.03 letters was obtained in the naïve patients. This is in line with previous findings: in the REBA study^17^, the naïve cohort reported a final mean gain of 11.9 ± 3.8 letters.

Functional improvement was noted also in switched patients, with a mean gain of 4.71 ± 6.06 letters. This is an encouraging finding: in fact, variable results were reported in long-term studies concerning BCVA. Haensli et al.^9^ showed that BCVA changed from 67.8 ± 7.2 at baseline to 72.2 ± 7.5 ETDRS equivalents after 6 months. In the REBA study^17^, a mean gain of 10.4 ± 4.8 letters after a minimum of 9 months of follow-up was demonstrated in switched patients. Abdin et al.^8^ showed a statistical visual improvement at week 16, then it remained stable compared to baseline until the end of the first year of treatment. This variability may be related to baseline anatomical status. Atrophic modifications may be induced both by AMD itself and by the treatment. A greater number of injections could be associated with the development of macular atrophy, and with the rate of its enlargement^28,29^.

Finally, we detected IOI in 4 out of 74 eyes (5.4%). In Literature, due to the variability in studies’ sample size, follow-up, and ethnicity, a large discrepancy among IOI rates after IVB was reported. In particular, 1 out of 19 treated eyes developed IOI in Montesel et al.^4^ cohort, 2 out of 21 eyes in Abdin et al.^8^ study, and 2 out of 7 eyes in Haensli et al.^9^ group; Japanese population seems to be at higher risk, with 15 cases in 68 treated eyes (22.1%) reported by Matsumoto et al.^7^. Adequate patient advertising and clinical monitoring are needed to avoid the occurrence of severe complications.

In conclusion, brolucizumab permits early fluid resolution in naïve patients, which could have significant implications for the preservation of anatomical integrity and visual function. Moreover, it works also in switched group, offering a new chance to refractory patients when other anti-VEGF agents cannot work anymore. Its efficacy in anatomical restoration and functional improvement, together with the extension of the treatment interval, make this molecule a promising new strategy for nAMD management, which could reduce the treatment burden in a long-term perspective. IOI occurrence represents a considerable adverse effect that must be promptly diagnosed and treated; however, early intervention could limit the damage extent. Studies with longer follow-ups are required to better understand the long-term effects of brolucizumab, focusing also on the meaning of PED dimensions’ variation and geographic atrophy incidence.

## Data Availability

The data that support the findings of this study are available from the corresponding author upon reasonable request.
